# The Association Between the China's Economic Development and the Passing Rate of National Physical Fitness Standards for Elderly People Aged 60–69 From 2000 to 2020

**DOI:** 10.3389/fpubh.2022.857691

**Published:** 2022-03-11

**Authors:** Zeyong Liu, Agudamu, Te Bu, Selcuk Akpinar, Blazo Jabucanin

**Affiliations:** ^1^College of Physical Education, Hunan Normal University, Changsha, China; ^2^Graduate School of Social Welfare, Sungkyunkwan University, Seoul, South Korea; ^3^Faculty of Sports Science, Nevşehir Haci Bektaş Veli University, Nevşehir, Turkey; ^4^Western Balkan Sport Innovation Lab, Podgorica, Montenegro

**Keywords:** GDP, population aging, public financing, public health, sports industry, policy coordination, spillovers

## Abstract

**Objective:**

According to the seventh demographic census, China's elderly population reached 260 million, accounting for 18.7% of the total population, indicating that China is on the verge of transitioning from a relatively mild aging to a moderately aging society, and an aging society inevitably brings concerns about the elderly people's health. The purpose of this study was to better understand the effect of economic development on the physical fitness of the elderly people aged 60–69 in China during the first two decades of the twenty-first century, as well as to establish a correlation between China's gross domestic product (GDP) and changes in the elderly people's passing rate of national physical fitness standards.

**Methods:**

A linear regression analysis was performed on the data of GDP and the passing rate of national physical fitness standards of Chinese elderly people aged 60–69 in 2000, 2005, 2010, 2014, and 2020.

**Results:**

The passing rate of national physical fitness standards for elderly people aged 60–69 increased linearly (*R*^2^ = 80.56%, *p* < 0.05), indicating that the physical fitness of the elderly tends to increase steadily with GDP expansion.

**Conclusions:**

Between 2000 and 2020, the annual improvement in the physical fitness of the elderly people in China is inextricably linked to rapid economic development. Increased financial investments in public sports services and a corresponding national fitness plan all contribute to an overall improvement in the physical fitness of the elderly people. This outcome is the effect of fiscal and policy coordination, which may represent a distinctive Chinese model and contribution to the global effort to manage and improve population physical fitness.

## Introduction

Physical fitness can be broadly defined as the state of being physically capable of performing one's daily tasks as well as performing at a satisfactory level in sports (1). Physical fitness is associated with morbidity and mortality and is also highly associated with health outcomes (2). Thus, physical fitness is critical for healthy life in all age groups. Due to the age-associated decline in physical fitness (3), it is critical to enhance involvement in physical activity and related physical fitness, particularly among the elderly people (4). Diets (5), cigarette smoking (6), and genetics (7) all function as mediators of an individual's physical fitness, and the economic environment has an effect on the population's level of physical fitness. It has been found that children and adolescents with a lower socio-economic status engage in less physical exercise and thus have lower levels of physical fitness than children with a higher socio-economic position (8). Similarly, it was discovered through secondary analysis of health and fitness survey data from 15 European countries that lower-income countries had lower levels of fitness in persons aged 50 and above when compared to higher-income countries (9). Increased economic wellbeing among the population may boost opportunities for physical activity, hence increasing physical fitness levels. Given that economic status might be a barrier for the elderly people, it would be quite advantageous to assist them in this area. It is a well-known fact that the world's elderly population is growing in many countries (10). One of those countries is China.

Between 2000 and 2020, China's society accelerated its aging. China's old population over the age of 60 reached 260 million in the seventh demographic census, accounting for 18.7% of the overall population (11). This shows that China is on the verge of transitioning from a relatively mild aging to a moderately aging society, and the resulting physical fitness of the elderly population becomes critical. To gain a comprehensive understanding of the national physical fitness situation, assist in completing the assessment of the national fitness plan, and supervise and promote the construction of a healthy China, the General Administration of Sport of China and the Ministry of Education and other relevant departments began cooperating in 2000.

The passing rate for elderly people's physical fitness standards was 86.3% in the 2000 National Physical Fitness Monitoring Bulletin (12). In the 2005, Second National Physical Fitness Monitoring Bulletin (13), a total of 27,125 elderly people were surveyed, and the passing rate for elderly people's physical fitness standards was 84.4%, a decrease of 1.9% from 2000. The 2010 National Physical Fitness Monitoring Bulletin (14) examined 25,712 adults nationally and reported an elderly passing rate of 86.4%, a 2% improvement from 2005. The 2014 National Physical Fitness Monitoring Bulletin (15) was published in November 2015. The bulletin used stratified random whole-group sampling to poll the elderly people aged 60–69, and a total of 25,719 people were surveyed nationwide. The results indicated that 87.1% of the elderly people passed the national physical fitness standards, an increase of 0.7% over 2010. According to the Fifth National Physical Fitness Monitoring Bulletin (16), the proportion of elderly people who earned a passing rate of the national physical fitness standards was 91.4%, up 4.3% from 2014.

Stable public financing is a critical aspect in ensuring the long-term and coordinated development of public sports services in China. To encourage public sports services to gradually transition to a “sports-oriented and diversified” model, the government has enacted policies such as the “Interim Measures for Contracting the Budgets of Public Sports Services Under the State Sports Commission” and implemented a graded accountability system for public expenditure on sports, with sports expenditure gradually shifting from the state to a combination of the state and society (17). In 1995, China enacted the People's Republic of China's Sports Law (18), which stipulates unequivocally that people's governments at or above the county level should incorporate sports funding and capital construction funds into their financial budgets and capital construction investment plans, and gradually increase investment in public sports services as the national economy develops. It also establishes a legal framework for the formulation and improvement of public sports services.

Government investment in physical fitness programs for the elderly is critical for the overall health of the population. In developed countries, the United States, the United Kingdom, and South Korea all invest more than 1% of their gross domestic product (GDP) in sports, the Swiss government invests 4.3% of its budget in sports and recreation, and the Russian Federation's State Sports Fund receives 2% of its budget (19). Although China's public financial investment in public sports services has increased, the proportion of public financial investment in sports to national GDP remains low (Table 1) (20), and the proportion of financial expenditure on sports in other developed nations remains a gap.

**Table 1:** List of China's public financial investments in sports as a percentage of gross domestic product (GDP).

**Year**	**Public financial investments in sports (billion Chinese Yuan)**	**GDP (trillion Chinese Yuan)**	**Percentage of GDP (%)**
2000	8.16	9.921	0.08
2001	10.82	11.086	0.09
2002	14.18	12.171	0.11
2003	15.53	13.742	0.11
2004	17.38	16.184	0.11
2005	18.02	18.731	0.10
2006	21.57	21.943	0.10
2007	26.01	27.009	0.10
2008	33.27	31.924	0.10
2009	31.35	34.851	0.09
2010	33.42	41.211	0.08
2011	36.52	48.794	0.07
2012	38.84	53.858	0.07
2013	31.58	59.296	0.05
2014	33.38	64.356	0.05
2015	35.50	68.885	0.05
2016	40.66	74.358	0.05
2017	46.90	82.075	0.06

There have been few research published to date examining the association between national GDP and physical activity levels, and the current evidence is conflicting. According to data collected from the 27 European Union member states, there is a statistically significant correlation (regression coefficient, 0.599; *p* < 0.10) between GDP and leisure-time physical activity (measured indirectly *via* surveys) (21). Another research of 76 nations discovered that more economically developed and urbanized countries (indirectly measured *via* the Human Development Index) have a higher proportion of their population (aged 15 and above) that does not get enough physical activity (measured indirectly *via* surveys) (22). Whereas, another study of 38 nations found a negative correlation between GDP and population physical activity (measured indirectly *via* surveys), with the highest levels of physical activity occurring in countries with a relatively low GDP (23). In short, there are no conclusive findings about the relationship between economic environment and physical activity levels or fitness, and there is no data on the elderly population.

China's economy has made significant achievement since the beginning of the twenty-first century, growing at an average annual rate of more than 8.65% from 2000 to 2020 (24). The GDP steadily increased from 10.028 trillion Chinese Yuan in 2000 to 101.599 trillion Chinese Yuan in 2020 (24). Meanwhile, as China's population ages, future risks related with demographic disorders, economic volatility, and an increase in chronic diseases are possible. When it comes to addressing the social issue of how to improve the physical fitness of the elderly people, more rigorous and comprehensive planning is required in order to promote the elderly people's physical fitness while encouraging the synergistic development of China's economy.

There is currently no research on the relationship between national economic development and physical fitness among China's elderly people. As a result, this study sought to determine this correlation in order to gain a better understanding of their relationship and to influence future national policies aimed at promoting the sustainable development of both the elderly population's physical fitness and economic policy in China.

## Methods

Between 2000 and 2020, GDP data for China Mainland were retrieved from the World Bank (24). The national physical fitness standards for elderly people aged 60–69 were collected from the National Physical Fitness Monitoring Bulletin to determine the passing rate. In China, national physical fitness standards are explored in depth using a multi-category comprehensive index capable of efficiently assessing the development of physical condition (25). The index system includes a total of 20 indications divided into three categories: body morphology, body function, and physical function, with six to nine indicators chosen for each category based on the physical features of various age groups of people. Indicators of body morphology represent the state of development of the human body, including body shape, posture, and nutritional status. Height, body weight, chest circumference, waist circumference, hip circumference, skinfold thickness (i.e., triceps, scapula, and abdomen), and body fat percentage are all used as body morphology markers in national physical fitness monitoring of elderly people aged 60–69. To address the needs of a broad national sample, the national physical fitness monitoring program can utilize only a few straightforward and easy-to-use body function indicators. The national physical fitness monitoring program for elderly people aged 60–69 uses blood pressure, resting heart rate, lung capacity, and a 2-min high knees as measures of body function. Physical function indicators refer to the ability of the human body to display speed, strength, agility, balance, and flexibility in sports. The national physical fitness monitoring program for elderly people aged 60–69 includes indicators for forward bend test in sitting position, grip strength, single-leg stance test with eyes closed, choice reaction time test, and 30-s of sit to stand test. The standards use the individual indicators' physical fitness test scores, followed by a holistic assessment of all indicators, to classify an individual's physical fitness within the same population into four categories: excellent, good, pass, and fail.

Due to the fact that the linear function gave the same results as the first-order polynomial function, linear regression was employed to investigate the association between GDP and the passing rate of national physical fitness standards. Pearson's correlation coefficient was used to quantify the relationship between the total value of GDP and the elderly people's passing rate in the five corresponding years: 2000, 2005, 2010, 2014, and 2020. The obtained linear regressions enabled us to forecast trend changes in the elderly people's physical fitness. *p* < 0.05 was considered statistically significant. The analyses in this work were conducted using prism 9 (GraphPad Software, San Diego).

## Results

Between 2000 and 2020, five national physical fitness monitoring bulletins were published. Tables 2–4 present a summary of single indicators of national physical fitness standards in 2010, 2014, and 2020. Individual indicators of physical fitness are generally above the passing norm, with the overall passing rate of national physical fitness standards for elderly people ranging from 86.3% in 2000 to 84.4% in 2005, 86.6% in 2010, 87.1% in 2014, and 91.4% in 2020.

**Table 2:** Indicators of body morphology average test results in national physical fitness standards.

**Sex**	**Year**	**Age group (yrs)**	**Height (cm)**	**Body weight (kg)**	**Chest circumference (cm)**	**Waist circumference (cm)**	**Hip circumference (cm)**	**Skinfold thickness (mm)**			**Body fat percentage (%)**
								**triceps**	**scapula**	**abdomen**	
Male	2000	60–64	165.4	64.5	89.5	83.0	92.6	12.3	17.7	21.9	/
		65–69	164.8	63.7	89.0	83.4	92.5	12.2	17.5	21.5	/
	2005	60–64	165.3	65.0	90.5	83.9	92.1	11.3	17.4	21.3	/
		65–69	165.0	63.9	89.8	83.5	91.9	11.5	17.2	20.9	/
	2010	60–64	165.7	66.6	91.5	85.4	93.0	11.0	16.8	20.6	/
		65–69	164.9	65.3	91.2	85.3	92.8	11.2	16.6	20.3	/
	2014	60–64	166.1	67.6	92.6	87.0	94.2	12.0	17.6	22.2	/
		65–69	165.4	66.6	92.2	86.8	94.0	12.3	17.5	21.9	/
	2020	60–64	165.9	69.0	/	89.3	96.4	/	/	/	23.3
		65–69	165.4	68.1	/	89.3	96.3	/	/	/	23.3
Female	2000	60–64	154.2	57.6	87.8	81.5	93.5	18.8	21.6	28.7	/
		65–69	153.4	56.5	87.1	81.8	93.0	17.9	20.3	27.4	/
	2005	60–64	154.1	58.0	88.4	82.9	93.2	19.8	21.9	28.9	/
		65–69	153.2	56.4	86.8	82.4	92.3	18.8	20.8	27.9	/
	2010	60–64	155.3	59.7	90.6	84.7	94.7	19.8	21.2	27.4	/
		65–69	154.4	59.2	90.4	85.5	94.5	19.5	20.8	27.0	/
	2014	60–64	154.5	59.2	89.5	84.0	94.1	20.0	21.1	26.9	/
		65–69	153.4	57.7	88.6	84.0	93.4	19.2	20.0	25.9	/
	2020	60–64	155.1	60.3	/	85.5	95.3	/	/	/	32.9
		65–69	154.4	59.8	/	86.4	95.4	/	/	/	33.0

**Table 3:** Indicators of body function average test results in national physical fitness standards.

**Sex**	**Year**	**Age group (yrs)**	**Systolic blood pressure (mm Hg)**	**Diastolic blood pressure (mm Hg)**	**Resting heart rate (beats·min^**−1**^)**	**Lung capacity (mL)**	**2-min high knees (times)**
Male	2000	60–64	130.7	81.2	78.1	2649.9	/
		65–69	132.8	80.9	78.5	2441.3	/
	2005	60–64	129.2	80.5	77.4	2,565	/
		65–69	131.1	79.6	77.3	2,364	/
	2010	60–64	130.2	81.6	76.0	2,611	/
		65–69	132.0	81.2	75.7	2,407	/
	2014	60–64	129.4	81.0	77.2	2,563	/
		65–69	131.0	80.2	76.8	2,432	/
	2020	60–64	/	/	/	2,509	51.8
		65–69	/	/	/	2,342	50.7
Female	2000	60–64	129.2	79.5	77.9	1861.0	/
		65–69	131.1	79.3	78.2	1761.6	/
	2005	60–64	127.6	78.2	76.8	1,725	/
		65–69	129.5	77.9	77.3	1,617	/
	2010	60–64	126.8	77.7	76.4	1,829	/
		65–69	129.5	78.0	76.8	1,747	/
	2014	60–64	128.1	79.4	75.6	1,766	/
		65–69	130.4	79.2	75.6	1,645	/
	2020	60–64	/	/	/	1,785	55.1
		65–69	/	/	/	1,679	52.3

**Table 4:** Indicators of physical function average test results in national physical fitness standards.

**Sex**	**Year**	**Age group (yrs)**	**Forward bend test in sitting position (cm)**	**Grip strength (kg)**	**Single-leg stance test with eyes closed (s)**	**Choice reaction time test (s)**	**30-s of sit to stand test (times)**
Male	2000	60–64	3.4	37.4	12.0	0.7	/
		65–69	1.6	34.9	9.4	0.8	/
	2005	60–64	1.9	37.5	10.8	0.66	/
		65–69	0.5	35.0	8.7	0.72	/
	2010	60–64	1.8	37.4	10.1	0.7	/
		65–69	0.5	34.6	8.2	0.7	/
	2014	60–64	2.1	37.3	9.4	0.67	/
		65–69	1.5	35.0	8.2	0.70	/
	2020	60–64	2.4	36.5	11.3	0.72	12.0
		65–69	1.7	35.1	10.3	0.74	11.7
Female	2000	60–64	8.4	23.9	9.7	0.8	/
		65–69	7.1	22.8	8.4	0.8	/
	2005	60–64	7.8	23.5	9.1	0.73	/
		65–69	6.4	22.2	7.9	0.78	/
	2010	60–64	7.9	23.2	8.5	0.71	/
		65–69	7.2	22.3	7.7	0.75	/
	2014	60–64	7.9	23.3	9.2	0.7	/
		65–69	6.7	21.8	7.4	0.8	/
	2020	60–64	7.9	22.8	109	0.74	11.8
		65–69	7.1	21.6	9.9	0.78	11.3

**Figure 1:**
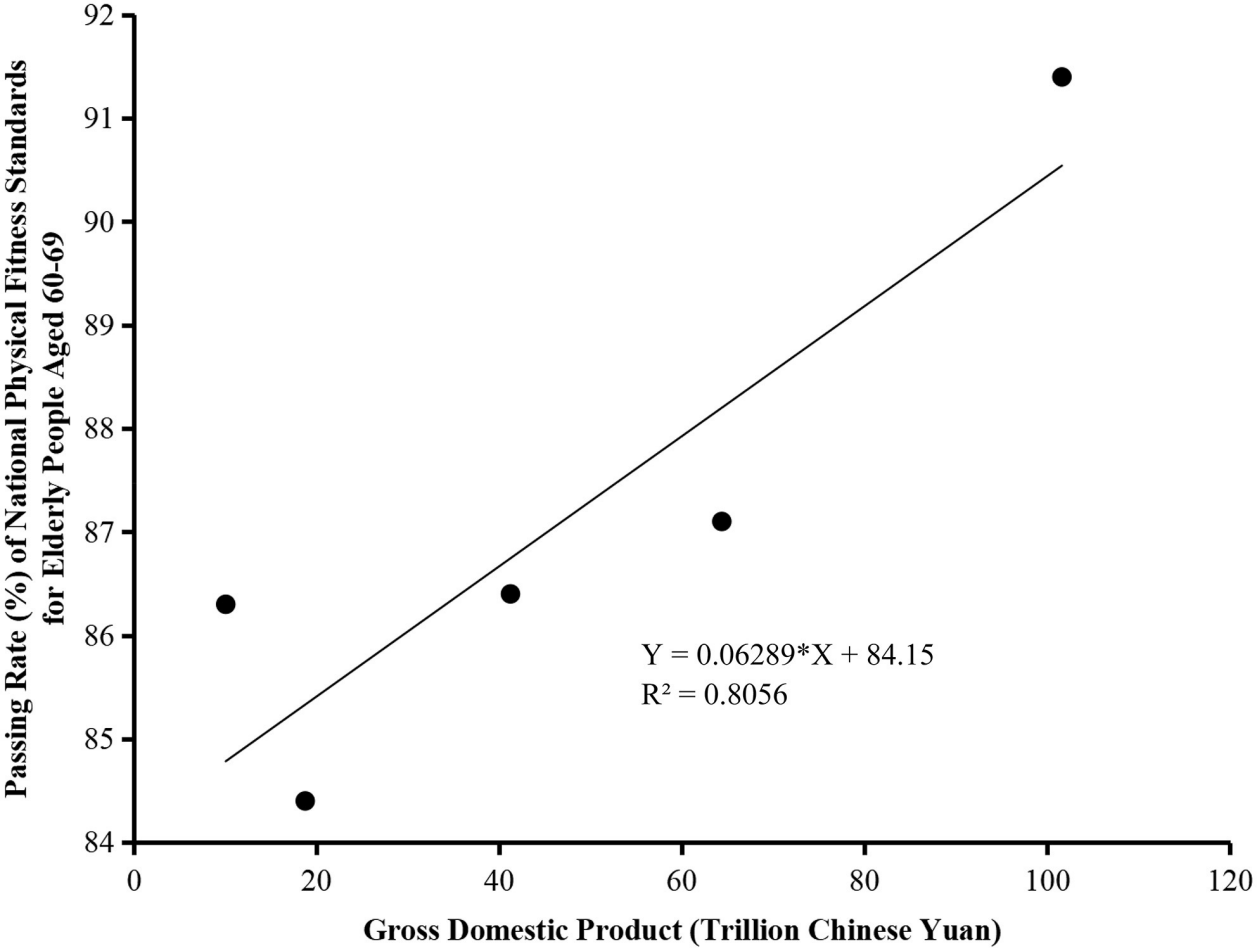
Linear regression of gross domestic product and elderly people's passing rate of national physical fitness standards in the five corresponding years: 2000, 2005, 2010, 2014, and 2020.

The correlation coefficient model is summarized in Figure 1. The Pearson correlation coefficient between the total value of GDP and the elderly people's passing rate of national physical fitness standards is 0.8976, indicating a positive association between the two. With a significant *p*-value of 0.0388, it can be concluded that GDP has a positive significant effect on the elderly people's passing rate of national physical fitness standards.

## Discussion

This is the first study in China to demonstrate a strong positive correlation between the total value of GDP and the passing rate of national physical fitness standards among the elderly in the corresponding year. China's average GDP growth rate over the last two decades has been as high as 9%, and the overall GDP has increased by nearly 1,085% (24). Rapid economic development has resulted in the development of health care and public sports services, laying the groundwork for the elderly people's physical fitness to continue to improve year after year.

Previous research on the relationship between economic development and physical fitness has primarily relied on indirect measures of the economic environment (e.g., Human Development Index) or indirect measures of physical fitness (e.g., surveys). This is the only study we are aware of that establishes a direct association between national economic development and large-scale, population physical fitness by measuring each activity directly (i.e., GDP and actual physical fitness tests). Particularly, lower body strength and grip strength, which are included in national physical fitness standards, are closely correlated with frailty, an age-related condition characterized by a vulnerability status that impairs the elderly people's quality of life and independence (4). Thus, the current findings provided the first direct analysis demonstrating how rapid economic expansion has impacted the physical fitness of the elderly population in the world's second largest economy.

China's financial expenditure on public sports services increased from 8.16 billion Chinese Yuan in 2000 to 46.90 billion Chinese Yuan in 2017 (20), an increase of approximately 5.75 times in 15 years. The absolute scale of financial expenditure on public sports services has steadily increased, but a stable growth mechanism has not yet been established. From 2000 to 2015, China's financial expenditure on public sports services hit its peak in 2012 (Table 1). However, it decreased by 19.7% in 2013 and by 5.9% following the 2008 Beijing Olympic Games, indicating that China's financial expenditure on public sports services has been impacted by the Olympic Games and other major international sports events. China spent 145.61 billion Chinese Yuan on public sports services during the 11th Five-Year Plan period (2006–2010), and 175.82 billion Chinese Yuan on public sports services during the 12th Five-Year Plan period (2011–2015) (20), an increase of 21% over the 11th Five-Year Plan period. Meanwhile, China's overall fiscal expenditure for the 11th Five-Year Plan period (2006–2010) was 31,897.08 billion Chinese Yuan, and during the 12th Five-Year Plan period (2011–2015), the total fiscal expenditure was 70,306.71 billion Chinese Yuan (20), a 220% increase over the 11th Five-Year Plan period. Thus, it can be observed that sports expenditures are growing at a slower rate than fiscal expenditures. However, as financial investment in public sports services increases year after year, China continues to establish comprehensive circumstances for physical fitness and exercise for the elderly population, while simultaneously improving the state of the public sports and fitness environment.

China's old population has been growing since the millennium's turn. The government's year-on-year increase in public funding on sports is certain to boost the elderly population's physical fitness. Under the policy framework of the National Fitness Plan (26), the Chinese government has gradually improved public sports and fitness facilities at three levels—counties (cities and districts), townships (streets), and administrative villages (communities)—and has increased support for senior sports clubs and other related organizations to provide practical assistance in improving the elderly population's physical fitness. Meanwhile, the General Administration of Sport of China, in collaboration with the National Office for the Aging and the China Senior Citizens Sports Association, has organized a series of more influential senior sports activities in China, including the National Senior Citizens Sports and Fitness Conference and group fitness and recreation activities. Government-sponsored sports events are rigorously developed to serve as a model for elderly population's engagement in physical fitness and sports (27). In the summer, more than 70% of elderly population in North, East, South Central, Southwest, and Northwest China engage in physical activity, and more than 60% of elderly population in North, Northeast, East, and South Central provinces engage in physical activity more than five times per week for a total of 30–60 min each session (28). Under the guidance of the National Fitness Plan (26), various departments in China have provided strong support for the elderly population's fitness, including instruction and teaching, fitness venues, medical protection, and activity organization, and have vigorously promoted the elderly population's active participation in sports and fitness activities, as well as made concerted efforts in a variety of areas to assist the elderly in improving their physical fitness.

China's governmental financial expenditure on sports has increased year after year in lockstep with GDP development, ensuring both a continuous increase in the pace at which physical fitness standards for the elderly population in China are passed and a high level of convenience for the elderly people to exercise and stay healthy. While China still lags behind other countries in terms of state expenditure on public sports services (19), it has achieved substantial outcomes and assisted the elderly people in developing their physical health in accordance with China's national development. In the future, China's state expenditure on public sports service shall continue to grow at a steady rate consistent with the national circumstances, and could significantly support the elderly people's ability to exercise and enhance their physical fitness.

We should caution that, current findings in China as a result of fiscal and policy coordination, may not necessarily be extrapolated to other countries, i.e., economic growth does not always result in an increase in population physical fitness. This phenomenon has been documented in previous research, where a negative correlation exists between GDP and levels of physical activity (22, 23). This discrepancy may be explained by the fact that China is a socialist country, which means that national policy is highly regulated and has a substantial impact on social and economic outcomes. Following the National Fitness Plan (26), the Chinese government establishes national physical fitness monitoring centers and exercise guidance stations throughout the country, utilizes big data to expand population-wide physical fitness monitoring, and develops local fitness exercise programs for the elderly people based on regional characteristics. Additionally, the Chinese government expands training and allocation of social sports management personnel and establishes a network of sports and fitness volunteers around the country to provide scientific counsel and help to the elderly population regarding fitness and exercise. These socialist-leaning policies ensure that public financial investments in sports reach the elderly population effectively. On the other hand, countries characterized by a free market economy may not achieve the same level of fiscal and policy coordination as China (29). Over the last four decades, China has enacted a series of fundamental market reforms to open up trade routes and investment flows, ultimately lifting hundreds of millions of family out of poverty and into the middle class. Its economic expansion is unlikely to be replicated by any country, both in terms of relative growth rate and absolute base effect. Likewise, other countries are less likely to emulate its centralized government policies. This steady rise in physical fitness among the Chinese elderly is a result of both impressive economic development and an efficient policy transmission channel, an unique Chinese phenomena.

In conclusion, China's ongoing economic expansion in tandem with fiscal expenditures between 2000 and 2020 has resulted in the continual provision and support of fitness activity for the elderly people within the context of national physical fitness standards. Additionally, the continuous increase in the passing rate of physical fitness standards for the elderly people in China over the last two decades is due not only to the rapid growth of China's GDP and the continuous expansion of China's public expenditure on sports, but also to the elderly people's growing awareness of exercise as a result of state, society, and public media education. As a result of the National Fitness Plan (26), the proportion of elderly people participating in physical activity has grown massively in China. The percentage of elderly people participating in fitness activities has been increasing, as has the rate of elderly people passing national physical fitness standards. By examining the rate and pattern of China's population growth, it is clear that the China's population is beginning an accelerated aging trend. It has been projected that (30), China will have approximately 180 million people aged 65 and over in 2020, accounting for ~13% of the total population, and more than 310 million in 2035, accounting for ~22.3% of the total population. The increasing age of society's population, as well as the gradual slowing and fluctuation of China's economic growth, would have an effect on the elderly population's health and fitness. The nation's financial funding for public sports services must continue to rise in the future to keep pace with the aging population's growth and to ensure the elderly people's physical fitness.

## Data Availability Statement

The original contributions presented in the study are included in the article/supplementary material, further inquiries can be directed to the corresponding authors.

## Author Contributions

ZL, A, and TB: conceptualization and methodology. ZL: formal analysis. ZL and A: writing—original draft preparation. TB, SA, and BJ: writing—review and editing. ZL and TB: project administration. TB: funding acquisition. All authors have read and agreed to the published version of the manuscript.

## Funding

This work was supported by Key Project of Education Special Subject of Hunan Social Science Fund in 2020: From four-in-one to integrated physical education: Xi Jinping's Thought on School Physical Education in the New Era (JJ209662).

## Conflict of Interest

The authors declare that the research was conducted in the absence of any commercial or financial relationships that could be construed as a potential conflict of interest.

## Publisher's Note

All claims expressed in this article are solely those of the authors and do not necessarily represent those of their affiliated organizations, or those of the publisher, the editors and the reviewers. Any product that may be evaluated in this article, or claim that may be made by its manufacturer, is not guaranteed or endorsed by the publisher.
